# The Effect of Moral Judgment on Bystander Cooperation Behavior: The Role of Personal Force

**DOI:** 10.3390/bs15121699

**Published:** 2025-12-08

**Authors:** Xiaodan Xu, Yidie Lai, Juan Wang, Yang Liu, Ming Yu, Feng Zhang, Yan Xu

**Affiliations:** 1 School of Arts and Communication, Beijing Normal University, Beijing 100875, China; 2 State Key Laboratory of Cognitive Neuroscience and Learning, Beijing Normal University, Beijing 100875, China; 3 Department of Psychology, School of Humanities and Social Sciences, Beijing Forestry University, Beijing 100083, China; 4 School of Psychology, Shanghai Normal University, Shanghai 200234, China; 5 Psychological Research and Counseling Center, Southwest Jiaotong University, Chengdu 610031, China; 6 Beijing Key Laboratory of Applied Experimental Psychology, National Demonstration Center for Experimental Psychology Education (Beijing Normal University), Faculty of Psychology, Beijing Normal University, Beijing 100875, China

**Keywords:** utilitarianism, cooperative behavior, personal force

## Abstract

Background: While extensive research has examined the antecedents of utilitarian moral judgment, its subsequent social consequences remain less explored. Drawing on the moral reciprocal partner selection model and the moral intuition modular myopia hypothesis, this study investigates the impact of utilitarian moral judgment on bystander cooperation behavior and the moderating role of personal force. Objectives: This research aims to determine whether utilitarian moral judgments, compared to non-utilitarian ones, decrease bystander cooperation (Hypothesis 1), and whether this effect is more pronounced when the utilitarian judgment involves personal force (Hypothesis 2). Methods: Two progressive between-subjects experiments were conducted. Experiment 1 (N = 159) employed a single-factor design (utilitarian vs. non-utilitarian judgment) using a footbridge dilemma scenario and a trust task to measure cooperation. Experiment 2 (N = 346) utilized a 2 (judgment: utilitarian vs. non-utilitarian) × 2 (personal force: personal force vs. no personal force) factorial design, employing the same trust task. Results: In Experiment 1, bystanders invested significantly fewer tokens in the trust task after observing a utilitarian judgment compared to a non-utilitarian one. Experiment 2 revealed a significant main effect of moral judgment and a significant interaction between moral judgment and personal force. Simple effects analysis confirmed that the negative effect of utilitarian judgments on cooperation was stronger when personal force was involved. Conclusions: Utilitarian moral judgments reduce bystander cooperation compared to non-utilitarian judgments, and this reduction is more substantial when the judgment involves personal force. These findings highlight the interpersonal costs of utilitarian decision-making and underscore the importance of contextual features like personal force in understanding its social reception.

## 1. Introduction

Utilitarianism is a moral philosophy that judges actions based on their consequences, specifically aiming to maximize overall benefits or welfare (Bentham, 1983; Lazari Radek & Singer, 2017). A utilitarian moral judgment, therefore, approves actions that produce the greatest good for the greatest number, even when these actions might violate conventional moral norms or individual rights (J. Greene, 2013; Kahane et al., 2018). For example, in the classic “trolley problem,” a utilitarian would approve of diverting a trolley to kill one person instead of five, as this action maximizes net welfare by saving more lives. However, a non-utilitarian would reject diverting the trolley, arguing that people should respect everyone’s rights and obligations (Kahane et al., 2018). While a substantial body of research has examined the antecedent variables influencing utilitarian moral judgment—including cognitive, emotional, and contextual factors such as individual expectations (Sawaoka et al., 2014), intention inference (Young & Saxe, 2011), situational distance (Eyal et al., 2008), and the social class of the actor (Xu et al., 2020)—recent work has continued to enrich this domain. For instance, personality traits such as curiosity have been linked to utilitarian tendencies (Smillie et al., 2021). Emotional and cognitive mechanisms, including alexithymia and guilt, have also been shown to modulate moral decision-making (Wu et al., 2022; Gangemi et al., 2025). Moreover, a recent systematic meta-analysis reaffirms the dual-process interplay of intuition and deliberation in shaping moral judgments (Fahrenwaldt et al., 2025). Despite this progress, less is known about the social consequences of utilitarian judgment. Recent work has begun to explore these consequences, including how they influence perceptions of trustworthiness and cooperative behavior (Everett et al., 2016, 2018; Bostyn & Roets, 2017; Crockett et al., 2021; Myers & Everett, 2025).

This research is motivated by three core rationales. First, cooperation is a fundamental pillar of human survival and societal functioning (Decety et al., 2004), and understanding how moral judgments shape cooperative intentions is essential for explaining interpersonal dynamics. Second, existing cross-cultural evidence on the social consequences of utilitarianism remains limited, with most studies conducted in Western contexts; verifying these effects in non-Western settings (e.g., China) can test the universality of relevant theoretical models. Third, clarifying the contextual factors that moderate the link between utilitarian judgments and cooperation will provide a more nuanced account of moral psychology in social interactions.

The primary purpose of this study is to systematically investigate two key questions: (1) whether utilitarian moral judgments reduce bystanders’ willingness to cooperate compared to non-utilitarian judgments; (2) whether personal force serves as a boundary condition that amplifies this negative effect.

This research makes three distinct contributions. First, it extends the literature by shifting the focus from antecedents to the boundary conditions of utilitarian judgments’ social consequences, addressing a critical gap in current knowledge. Second, it integrates two complementary theoretical frameworks—the moral reciprocity theory (partner selection model) and the modular myopia hypothesis—to provide a comprehensive explanation for how and when utilitarian judgments influence cooperation. Third, by validating these effects in a Chinese cultural context, it enhances the cross-cultural generalizability of findings on moral judgment and social behavior, offering insights for fields ranging from psychology and philosophy to organizational behavior and policy-making.

## 2. Theoretical Framework and Hypotheses Development

To examine how utilitarian moral judgments affect bystanders’ willingness to cooperate, we draw on two complementary theoretical perspectives: the moral reciprocity theory (partner selection model) (Baumard et al., 2013; Barclay, 2016) and the modular myopia hypothesis (J. Greene, 2013). These theories were chosen because they offer complementary mechanisms—one focusing on the social signaling value of moral judgments in partner choice, and the other on the cognitive–emotional processes that make certain utilitarian judgments particularly aversive. Together, they provide a comprehensive basis for understanding the social consequences of utilitarian judgment.

### 2.1. Moral Reciprocity Theory and Bystander Cooperation

Moral reciprocity theory, particularly the partner selection model (Baumard et al., 2013; Barclay, 2016), suggests that humans enhance their adaptive capacity by establishing long-term, mutually beneficial relationships with trustworthy individuals. This theory proposes that people seek social partners who can maximize mutual benefits, but they are more inclined to select those who respect rights and obligations, as such partners are perceived as more beneficial and reliable in the long term (Everett et al., 2016). From this perspective, moral judgments serve as crucial signals about an individual’s potential reliability as a cooperative partner (Baumard et al., 2013).

In this research, we define “bystander cooperation” as the willingness of individuals to engage in cooperative behaviors with others after observing their moral judgments, consistent with operationalizations used in prior trust game and social dilemma research (e.g., Bostyn & Roets, 2017; Everett et al., 2018). Cooperation here refers to behaviors involving mutual exchange, shared resources, or joint efforts that could benefit both parties, such as sharing in economic games, collaborating on tasks, or forming alliances. This focus on bystander cooperation is critical because humans survive and thrive through cooperation (Decety et al., 2004). We must constantly decide with whom to cooperate.

The theoretical perspective of moral reciprocity explains why utilitarian judgments—which prioritize outcomes over individual rights—may signal an undesirable potential partner. By approving instrumental harm for the greater good, utilitarian decision-makers may be perceived as less committed to deontological norms that protect individuals from being used merely as means to an end (Everett et al., 2016). Previous research in Western contexts has confirmed this proposition, showing that participants were reluctant to trust and cooperate with utilitarian decision-makers (Bostyn & Roets, 2017; Everett et al., 2018; Rom et al., 2017). More recent cross-cultural work has further substantiated these findings, demonstrating reduced trust in utilitarian decision-makers across diverse populations (Everett et al., 2021). Based on moral reciprocity theory, we first examine the basic relationship between utilitarian judgment and bystander cooperation:

**Hypothesis** **1.** *Compared with non-utilitarian judgments, utilitarian judgments decrease bystander cooperation behavior.*

### 2.2. The Modular Myopia Hypothesis and the Role of Personal Force

To develop a more nuanced understanding of when this effect occurs, we incorporate the modular myopia hypothesis (J. Greene, 2013) into our framework. This hypothesis was chosen because it specifically explains why certain salient features of moral dilemmas—particularly personal force—elicit strong emotional responses that can override deliberative cost–benefit analysis, thereby influencing social evaluations. This hypothesis proposes that cognitive subsystems within the human brain automatically identify and monitor individuals’ planned actions. When an individual intends to harm another, it triggers an emotional alarm. Moral intuition, in response to this alarm system, rejects utilitarian judgments or decisions that elicit an emotional response. Crucially, this cognitive subsystem operates as a “single-channel” review mechanism that cannot simultaneously examine multiple causal chains leading to harm.

The modular myopia hypothesis explains why certain features of moral situations—particularly personal force—might intensify negative reactions to utilitarian judgments. Personal force refers to force exerted through the muscular movements of the actor, such as pushing someone off a footbridge with your hands to stop a trolley from killing five people. This is distinct from non-personal force, such as flipping a switch that diverts a trolley onto a track where it will kill one person instead of five (Cushman et al., 2006; J. D. Greene et al., 2009). Previous research has identified personal force as the critical factor distinguishing reactions between the footbridge and trolley dilemmas, as personal force triggers stronger emotional responses in the brain (Moore et al., 2008; Young et al., 2006).

Building on our first hypothesis and integrating the modular myopia hypothesis, we propose that the negative effect of utilitarian judgments on cooperation will be particularly pronounced when these judgments involve personal force. The direct, physical nature of personal force is likely to trigger more intense emotional alarms in bystanders, leading to stronger negative evaluations of the decision-maker’s character and trustworthiness (J. Greene, 2013).

**Hypothesis** **2.** *Utilitarian judgments involving personal force lead to a greater reduction in bystanders’ cooperative behaviors compared to utilitarian judgments without personal force.*

### 2.3. The Present Research

The present study systematically explores the impact of utilitarian judgments on bystanders’ cooperative behaviors through two progressive experiments. Experiment 1 tests the basic proposition that utilitarian judgments decrease bystander cooperation compared to non-utilitarian judgments (Hypothesis 1). Experiment 2 builds on this foundation to examine whether personal force serves as a boundary condition moderating this effect (Hypothesis 2). This approach allows us to first establish whether the basic effect exists and then determine the specific conditions under which it is most pronounced.

By understanding these dynamics, we can gain insight into how moral judgments influence social cooperation—a fundamental aspect of human interaction with implications for various fields including psychology, philosophy, economics, and organizational behavior.

## 3. Experiment 1

### 3.1. Purpose and Hypothesis

Experiment 1 aimed to test Hypothesis 1, which proposed that utilitarian moral judgments decrease bystander cooperation behavior compared to non-utilitarian moral judgments.

### 3.2. Method

#### 3.2.1. Participants

G*Power 3.1 software was used to calculate the minimum sample size (Faul et al., 2007). The analysis indicated that a sample of 128 participants was required to achieve a power of the test (1-β) of 0.8, a significance level of 0.05, and a medium effect size (*f* = 0.25). However, to ensure robustness and account for potential attrition, 159 Chinese participants (60 males, 99 females; *M*_age_ = 27.51, *SD*_age_ = 7.83) were recruited through Credamo (https://www.credamo.com/#/, accessed on 7 March 2023). Participants were required to be at least 18 years old and fluent in Chinese. Exclusion criteria included failing an attention check item. The participants were randomly assigned to either the utilitarian moral judgment group (n = 77) or the non-utilitarian moral judgment group (n = 82). Upon completion of the study, each participant received compensation of 1 CNY.

#### 3.2.2. Experimental Design

A single-factor between-subjects design was employed, with utilitarian moral judgment vs. non-utilitarian moral judgment as the independent variable. The number of tokens invested in a trust task served as the dependent variable.

#### 3.2.3. Experimental Materials and Tools

Manipulation of moral judgment: We adapted the footbridge dilemma from Everett et al. (2018). Participants read a scenario in which an agent (“Person A”) expressed either a utilitarian or non-utilitarian judgment regarding whether a character should push a stranger off a footbridge to save five workers (see Table 1 for the full scenario and justifications).

Manipulation Check: As a manipulation check, participants were asked to indicate person A’s position regarding what Zhang Li should do. For example, they selected between the following two statements: “Zhang Li should push the stranger who happens to be large to save the five workers”, or “Zhang Li should not push the stranger who happens to be large to save the five workers.” Participants who answered correctly proceeded to the next phase of the experiment, while those who answered incorrectly were excluded from analysis.

Measurement of Bystander Cooperation Behavior: We used a trust task adapted from Bostyn and Roets (2017). Participants acted as investors and were endowed with 10 tokens to invest in Partner A. Each token invested was tripled for Partner A, who could then return any number of tokens to the participant. The amount invested served as the behavioral measure of cooperation.

Attention Check: An attention check item was embedded in the questionnaire (e.g., “Please select ‘4’ for this item”). Participants who failed this check were excluded from the analysis.

Measurement of Subjective Social Status: We measured subjective social status with the MacArthur Scale of Subjective Social Status (Adler et al., 2000). Participants were asked to identify their position on a 10-rung ladder representing societal standing, with the bottom rung (1) signifying the lowest status and the top rung (10) the highest.

#### 3.2.4. Experimental Procedure

The experiment was conducted online via the Credamo platform. After providing informed consent, participants completed the following procedure in sequence:

Baseline Assessment: Participants first completed an initial trust task to establish a benchmark for their behavioral tendencies.

Random Assignment: A between-subjects design was used to randomly assign participants to one of two conditions: utilitarian judgment group (n = 77) or non-utilitarian judgment group (n = 82).

Moral Judgment Manipulation: Participants read the footbridge dilemma scenario (Table 1) where “Person A” (their upcoming partner) expressed either a utilitarian or non-utilitarian judgment about whether Zhang Li should push the stranger off the bridge.

Cooperation Measurement: Participants engaged in the trust task with Person A, deciding how many of their 10 tokens to invest, which served as our measure of bystander cooperation behavior.

Demographic information: Finally, participants completed questionnaires assessing gender, age, and subjective social status, which were used as covariates in the analysis.

The entire procedure took approximately 15 min to complete.

#### 3.2.5. Data Analysis

All statistical analyses were performed using IBM SPSS version 25.0. Prior to conducting the main analyses, we assessed the normality of the dependent variable using the Shapiro–Wilk test. The results indicated that the data did not significantly deviate from normality (*p* > 0.05), supporting the use of parametric tests.

A one-way analysis of variance (ANOVA) was first conducted to compare the baseline trust task scores between the two experimental groups, ensuring that no pre-existing differences existed prior to the manipulation.

To test the main hypothesis, an analysis of covariance (ANCOVA) was performed. This test was selected to control for the potential influence of covariates—gender, age, and subjective social status—on the dependent variable. The independent variable was the type of moral judgment (utilitarian vs. non-utilitarian), and the dependent variable was the number of tokens invested in the trust task after the moral judgment manipulation.

Effect sizes are reported as partial eta-squared (η^2^), and the significance level was set at α = 0.05.

### 3.3. Results

A one-way ANOVA revealed that no significant difference in the number of tokens invested during the baseline measurement of the trust task between participants in the utilitarian judgments group (*M* = 5.94, *SD* = 2.89) and the non-utilitarian judgment group (*M* = 5.79, *SD* = 2.77), *F*(1, 157) = 0.10, *p* = 0.75, $\eta_{p}^{2}$ = 0.001.

#### 3.3.1. Main Effect

**Figure 1:**
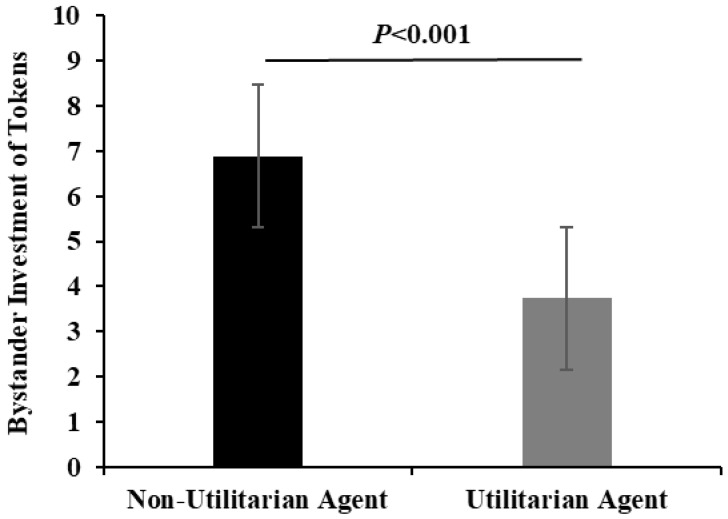
The effects of moral judgment on the number of tokens invested in the bystander trust task.

After controlling for gender, age, and subjective social status, an ANCOVA indicated that the number of tokens invested in the trust task by bystanders in the utilitarian moral judgment group (*M* = 3.73, *SD* = 3.47) was significantly lower than in the non-utilitarian moral judgment group (*M* = 6.89, *SD* = 2.53), *F* (1, 157) = 41.71, *p* < 0.001, $\eta_{p}^{2}$ = 0.21; see Figure 1.

#### 3.3.2. Brief Discussion of Findings

The results of Experiment 1 provide strong initial support for Hypothesis 1. The significant reduction in tokens invested by bystanders who observed a utilitarian judgment, compared to those who observed a non-utilitarian one, indicates that utilitarian moral judgments can negatively impact subsequent cooperative behavior. This finding aligns with the moral reciprocity theory, suggesting that individuals perceive utilitarian decision-makers as less desirable cooperation partners, possibly due to a perceived disregard for individual rights in favor of aggregate outcomes. These results establish a foundational effect for further investigation into its boundary conditions.

## 4. Experiment 2

### 4.1. Purpose and Hypothesis

Experiment 2 built on the findings of Experiment 1 by testing Hypothesis 2, which proposed that utilitarian judgments involving personal force would lead to a greater reduction in bystanders’ cooperative behaviors. This experiment employed a 2 × 2 factorial design to examine the moderating role of personal force.

### 4.2. Method

#### 4.2.1. Participants

The G*power 3.1 software was also used to calculate the minimum sample size required to detect a two-way interaction effect (Faul et al., 2007), which indicated a requirement of 231 participants. A total of 346 participants (225 females; *M*_age_ = 29.40, *SD*_age_ = 8.45) were recruited through Credamo (https://www.credamo.com/#/, accessed on 19 March 2023). The same inclusion and exclusion criteria as in Experiment 1 were applied.

#### 4.2.2. Experimental Design

A 2 (agent judgment: utilitarian vs. non-utilitarian) × 2 (personal force: present vs. absent) between-subjects experimental design was employed. The dependent variable was the number of tokens invested by the participants in the trust task. All participants were randomly assigned to four groups: utilitarian–personal force group (n = 88), utilitarian–no personal force group (n = 85), non-utilitarian–personal force group (n = 88), and non-utilitarian–no personal force group (n = 85).

#### 4.2.3. Experimental Materials and Tools

Moral Judgment and Personal Force Manipulation: We used scenarios adapted from Everett et al. (2018) and Bago et al. (2022). The personal force condition involved pushing a stranger, while the no-personal-force condition involved flipping a switch to drop the stranger (see Table 2 for full details).

Manutiplation Check: same as in Experiment 1.

Measurement of Bystander Cooperation Behavior: The trust task, adapted from Bostyn and Roets (2017), was applied to examine bystander cooperation behavior, replicating the procedure from Experiment 1.

Attention Check: The attention check was implemented in the same way as in Experiment 1.

#### 4.2.4. Experimental Procedure

The experimental procedure followed the same sequence as Experiment 1, with modifications to accommodate the 2 × 2 factorial design:

Baseline of Trust Task: Identical to Experiment 1, participants first completed the baseline trust task.

Random Assignment: Participants were randomly assigned to one of four experimental conditions in the 2 (moral judgment: utilitarian vs. non-utilitarian) × 2 (personal force: present vs. absent) between-subjects design.

Experimental Manipulation: Participants were presented with one of four scenario variations (Table 2) that crossed the moral judgment type (utilitarian vs. non-utilitarian) with personal force manipulation (footbridge/push vs. switch/drop).

Cooperation Measurement: Identical to Experiment 1, participants completed the trust task with their assigned partner as a measure of cooperative behavior.

Demographic Information: Participants provided the same demographic information as in Experiment 1.

The procedure took approximately 15 min to complete. All experimental materials, procedures, and measures were identical to those in Experiment 1 except for the specific scenario manipulations.

#### 4.2.5. Data Analysis

All statistical analyses were performed using IBM SPSS version 25.0. Prior to conducting the main analyses, we assessed the normality of the dependent variable (number of tokens invested in the trust task) across all experimental groups using the Shapiro–Wilk test. The results indicated that the data in some groups significantly deviated from normality (*p* < 0.05), suggesting violations of the normality assumption required for parametric tests.

A two-way analysis of variance (ANOVA) was first conducted to compare the baseline trust task scores across the four experimental groups, ensuring no pre-existing differences prior to the manipulation.

To test the main and interaction effects, a two-way analysis of covariance (ANCOVA) was performed, controlling for gender, age, and subjective social status. This test was selected to examine the effects of the independent variables—moral judgment (utilitarian vs. non-utilitarian) and personal force (present vs. absent)—on the dependent variable, while accounting for potential confounding factors.

Following a significant interaction effect, simple effects analyses were conducted. Due to violations of normality in some groups, non-parametric Mann–Whitney U tests were used for pairwise comparisons between groups. This method is robust to non-normality and appropriate for continuous data that do not meet parametric assumptions.

Effect sizes are reported as partial eta-squared (η^2^) for ANOVA/ANCOVA, and the significance level was set at α = 0.05.

### 4.3. Results

Baseline Trust Task: A two-way ANOVA revealed a non-significant main effect of moral judgment *F*(1, 342) = 0.10, *p* = 0.75, $\eta_{p}^{2}$ = 0.00); a non-significant main effect of personal force, *F*(1, 342) = 0.27, *p* = 0.60, $\eta_{p}^{2}$ = 0.001; and no significant interaction between moral judgment and personal force, *F*(1, 342) = 0.19, *p* = 0.67, $\eta_{p}^{2}$ = 0.001.

#### 4.3.1. Main and Interaction Effects

After gender, age, and subjective social status were controlled, an ANCOVA indicated that the main effect of moral judgment was significant (*F*(1, 339) = 51.71, *p* < 0.01, $\eta_{p}^{2}$ = 0.13), but the main effect of personal force was not significant (*F*(1, 339) = 0.33, *p* = 0.56, $\eta_{p}^{2}$ = 0.001). In addition, as expected, the interaction between moral judgment and personal force was significant (*F*(1, 339) = 12.04, *p* < 0.01, $\eta_{p}^{2}$ = 0.03). Means and standard deviations are presented in Table 3.

#### 4.3.2. The Moderating Role of Personal Force

**Figure 2:**
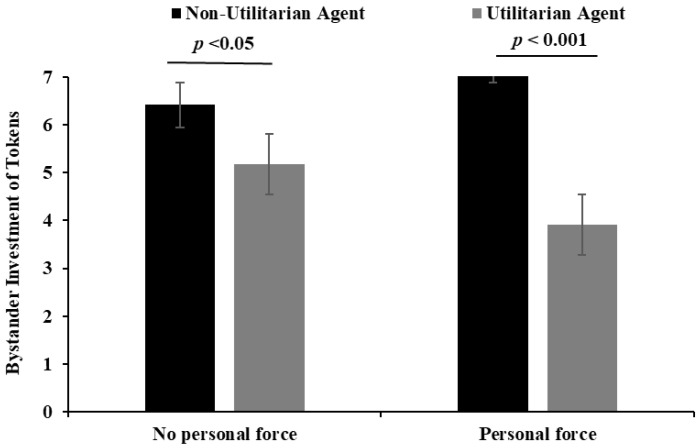
The effects of moral judgment and personal force on the number of tokens invested in the bystander trust task.

To decompose the significant interaction effect, simple effects analyses were planned. Since preliminary checks indicated that the data for the number of tokens invested were not normally distributed within some groups, we opted to use the nonparametric Mann–Whitney U test for these pairwise comparisons. This approach is robust to non-normality and is appropriate for ordinal or continuous data that do not meet parametric assumptions. When personal force was present, the number of tokens was significantly lower for subjects in the utilitarian group than for those in the non-utilitarian group (Mann–Whitney U = 1468, *p* < 0.001). When the subjects applied no personal force, the number of tokens was significantly lower for the subjects in the utilitarian group than for the subjects in the non-utilitarian group (Mann–Whitney U = 2838, *p* < 0.05) (see Figure 2).

#### 4.3.3. Brief Discussion of Findings

Experiment 2 successfully replicated the main negative effect of utilitarian judgments on cooperation found in Experiment 1 and, more importantly, identified a significant boundary condition. The significant interaction effect, followed by simple effects analyses, confirmed Hypothesis 2. The reduction in bystander cooperation following a utilitarian judgment was substantially stronger when that judgment involved personal force compared to when it did not. This pattern supports the modular myopia hypothesis, suggesting that direct, physical involvement in causing harm (personal force) triggers a more intense negative emotional and evaluative response in bystanders, thereby exacerbating the social cost of utilitarian decision-making.

## 5. General Discussion

This study investigated the effects of utilitarian judgments on bystanders’ cooperative behaviors and their boundary conditions through two experiments. The results indicated that, compared with non-utilitarian moral judgments, utilitarian moral judgments reduced bystanders’ cooperative behaviors (Experiment 1), and this effect was more pronounced when the judgments involved personal force (Experiment 2). These findings demonstrate that utilitarian judgments can produce significantly greater negative social consequences when they entail personal force.

### 5.1. The Role of Bystanders in Utilitarian Judgments

Utilitarian judgments convey important social signals (Haidt, 2001), which may influence bystanders’ social perceptions and behavioral interactions with the decision-maker. The theoretical foundation for understanding these bystander responses draws from the partner selection model of moral reciprocity (Baumard et al., 2013; Barclay, 2016). The theory suggests that moral judgments serve as indicators of an individual’s reliability as a potential cooperative partner, thereby influencing others’ willingness to engage in cooperative relationships.

Our research empirically investigates how utilitarian judgments affect bystanders’ cooperative behaviors toward the judgment agent in the Chinese cultural context. The findings demonstrate that Chinese participants, similar to their Western counterparts, exhibit reduced willingness to cooperate with individuals who make utilitarian moral decisions (consistent with studies by Bostyn & Roets, 2017; Everett et al., 2016, 2018; Rom et al., 2017; Everett et al., 2021). This cross-cultural consistency suggests a potentially universal tendency to view utilitarian decision-makers as less desirable social partners, aligning with partner-choice models of moral evolution (Baumard et al., 2013; Barclay, 2016; Crockett et al., 2021). Recent work further underscores the role of emotional processes in such evaluations, including how guilt and uncertainty influence moral decisions (Gangemi et al., 2025; Dahò, 2025).

A possible explanation for this phenomenon is that individuals who demonstrate respect for established norms, social structures, and duties are perceived as more beneficial and reliable interaction partners by bystanders (Everett et al., 2016). Such individuals may signal greater predictability and trustworthiness in social exchanges compared to those making purely outcome-based utilitarian calculations.

### 5.2. The Moderating Effect of Personal Force

This section examines how personal force moderates bystanders’ responses to utilitarian judgments of consequences among bystanders. Our investigation is theoretically grounded in J. Greene’s (2013) modular myopia hypothesis, which suggests that moral judgments are influenced by automatic emotional responses that are sensitive to certain situational features while potentially overlooking others. Our findings demonstrate significant moderating effects of personal force, revealing that utilitarian judgments more strongly reduce bystanders’ cooperative behaviors when they specifically involve personal force.

This pattern supports the modular myopia hypothesis, as bystanders appear to generate heightened emotional responses specifically in response to these salient moral features. Our findings are consistent with J. Greene’s (2013) modular myopia hypothesis, which emphasizes the role of automatic emotional alarms in moral cognition. This aligns with broader evidence that emotional and intuitive processes are fundamental to moral judgment (Fahrenwaldt et al., 2025) and that individual differences in traits such as psychopathy or alexithymia can alter moral response patterns (Wu et al., 2022). Personal force, as a salient situational feature, appears to amplify these underlying emotional and cognitive dynamics.

These findings align with previous research, which shows that individuals exhibit the lowest moral acceptability for utilitarian judgments involving personal force (J. D. Greene et al., 2009). Furthermore, our findings reveal that bystanders’ responses to utilitarian judgments demonstrate subjectivity and stereotyping (Yuan & Liu, 2021), as their cooperative behaviors are influenced not merely by the utilitarian calculation itself, but by the manner in which the utilitarian outcome is achieved. The reduction in cooperative behavior is not uniform across all utilitarian decisions but is particularly pronounced when the decision-maker employs personal force to cause harm to achieve the greater good.

By identifying these specific conditions under which utilitarian judgments most strongly affect social perceptions and cooperative behaviors, this research provides a more nuanced understanding of the relationship between utilitarian moral reasoning and its interpersonal consequences. This contribution extends our knowledge of how particular features of moral dilemmas modulate the social costs associated with utilitarian decision-making.

### 5.3. Theoretical and Practical Implications

#### 5.3.1. Theoretical Implications

This study makes two key contributions to moral psychology. First, it shifts the research focus from examining antecedents to systematically investigating the consequences of utilitarian judgments. Second, by establishing boundary conditions–specifically demonstrating that utilitarian judgments produce significantly stronger reductions in bystander cooperation when they involve personal force–we advance a more nuanced understanding of moral intuition’s role as an affective alarm system. These findings reveal that the social costs associated with utilitarian decision-making is systematically modulated by specific characteristics of the moral situation.

Our findings are well explained by, and extend, partner-choice models of moral psychology (Baumard et al., 2013; Barclay, 2016). These models posit that moral judgments serve as signals for identifying reliable cooperative partners. The robust reduction in cooperation we observed toward utilitarian agents aligns with this framework, suggesting that bystanders perceive a prioritization of aggregate outcomes over individual rights as a signal of unreliability in social exchange. This interpretation is strongly supported by a large body of work demonstrating that utilitarian decision-makers are consistently perceived as less trustworthy and are less preferred as social partners (Everett et al., 2016, 2018; Bostyn & Roets, 2017). Crucially, the cross-cultural consistency between our findings in a Chinese sample and previous Western findings (e.g., Everett et al., 2021) suggests this may be a fundamental aspect of human social cognition, potentially rooted in evolved partner-choice mechanisms.

Furthermore, our results concerning the moderating role of personal force provide critical nuance to this picture. The finding that the social penalty for utilitarianism is significantly amplified when harm involves personal force is elegantly accounted for by J. Greene’s (2013) modular myopia hypothesis. This theory suggests that moral intuitions are driven by an emotionally based alarm system that is particularly sensitive to salient, “up-close-and-personal” forms of harm. Our data confirm that this cognitive mechanism not only influences first-person moral judgments but also powerfully shapes third-person social perceptions, leading to a more severe cooperative penalty.

Our work also resonates with recent studies highlighting how personality and emotional granularity shape moral judgments (Smillie et al., 2021; Wu et al., 2022), and how clinical and emotional contexts modulate utilitarian decision-making (Dahò, 2025). Furthermore, the robust meta-analytic synthesis by Fahrenwaldt et al. (2025) provides a comprehensive backdrop for interpreting our results, confirming that moral judgments are driven by a complex interplay of intuitive and deliberative processes.

Finally, our work connects with the contemporary two-dimensional model of utilitarian psychology (Kahane et al., 2018; Everett & Kahane, 2020). This model dissociates utilitarian judgments into two dimensions: instrumental harm (endorsing harm for the greater good) and impartial beneficence (impartially maximizing welfare). Our study specifically implicates the instrumental harm dimension as the driver of reduced cooperation, a dimension consistently linked to negative social perceptions and reduced trust (Everett et al., 2018; Crockett et al., 2021). By identifying personal force as an intensifier of the social costs of instrumental harm, our research helps refine our understanding of the specific conditions under which different facets of utilitarian reasoning trigger social aversion.

#### 5.3.2. Practical Implications

Our findings offer important practical implications that refine our understanding of utilitarian decision-making’s social consequences. Contrary to generalized assumptions about utilitarianism’s uniformly negative social impact, our research reveals a more nuanced reality: the reduction in bystanders’ cooperative behaviors is significantly more pronounced when utilitarian judgments specifically involve personal force.

From a practical perspective, these results caution against overgeneralized moral evaluation of utilitarian decision-making. Instead, they advocate for more discerning assessment that considers the specific manner in which instrumental harm occurs. When evaluating utilitarian decisions in professional, organizational, or policy contexts, stakeholders should recognize that social responses may vary substantially depending on whether the decision-maker’s actions involve personal force.

### 5.4. Limitations and Future Directions

First, we examined the consequences of utilitarianism primarily from the perspective of moral judgment. Second, future studies should delve more deeply into the neural mechanisms underlying how utilitarian moral judgments influence bystanders’ cooperative behaviors. Thirdly, we employed standardized scenarios derived from the trolley dilemma and footbridge dilemma paradigms to systematically investigate how utilitarian judgments influence bystanders’ cooperative behaviors. While these well-established paradigms offer critical experimental control and enable direct comparisons with prior work (e.g., J. D. Greene et al., 2009), we acknowledge their inherent limitations in ecological validity. Our findings are context-dependent and should not be overgeneralized beyond the specific parameters of these controlled dilemmas. The artificial nature of such scenarios, though methodologically necessary, may not fully capture the complexity of real-world moral decision-making. Future research could explore more realistic moral dilemmas to investigate the influence of utilitarian judgments on bystanders’ cooperative behaviors.

## 6. Conclusions

This study not only highlights the importance of bystanders in utilitarian judgments but also identifies the specific conditions under which this agency becomes significant. Compared with non-utilitarian moral judgments, utilitarian moral judgments reduced bystanders’ cooperative behaviors, and this effect was more pronounced under conditions involving personal force. These findings enhance our understanding of the social consequences of utilitarian by identifying not only the general pattern of reduced cooperation but also the specific conditions under which this effect is most pronounced. This research contributes to a more nuanced understanding of utilitarian moral psychology by showing how the specific manner in which instrumental harm is implemented systematically shapes its social reception.

## Figures and Tables

**Table 1:** Agents’ judgments and justifications in Experiment 1.

	Non-Utilitarian Agent	Utilitarian Agent
A runway trolley is heading down the tracks toward five workers who will all be killed if the trolley proceeds on its present course. Zhang Li is on a footbridge over the tracks, in between the approaching trolley and the five workers. Next to him on this footbridge is a stranger who happens to be large. The only way to save the lives of the five workers is to push this stranger off the bridge and onto the tracks below, where his large body will stop the trolley. The stranger will die if Zhang Li does this, but the five workers will be saved. Should Zhang Li push the stranger to his death in order to save the five workers?	Person A (your matching partner) thinks that Zhang Li should not push the stranger who happens to be large off the bridge. The reason: I think that Zhang Li should not push the stranger who happens to be large off the bridge to save the five workers. I know that by doing this he could stop the trolley and save more lives, but I think that killing people is just wrong even if it has good consequences.	Person A (your matching partner) thinks that Zhang Li should push the stranger who happens to be large off the bridge. The reason: I think that Zhang Li should push the stranger who happens to be large off the bridge to save the five workers. By doing this, he could stop the trolley and save more lives, and I think that it is better to save many lives than just one life.

**Table 2:** Agents’ judgments and justifications in daily scenarios.

		Non-Utilitarian Agent	Utilitarian Agent
**Personal force**	A runway trolley is heading down the tracks toward five workers who will all be killed if the trolley proceeds on its present course. Zhang Li is on a footbridge over the tracks, in between the approaching trolley and the five workers. Next to him on this footbridge is a stranger who happens to be large. The only way to save the lives of the five workers is to push this stranger off the bridge and onto the tracks below, where his large body will stop the trolley. The stranger will die if Zhang Li does this, but the five workers will be saved. Should Zhang Li push the stranger to his death in order to save the five workers?	Person A (your matching partner) thinks that Zhang Li should not push the stranger who happens to be large off the bridge. The reason: I think that Zhang Li should not push the stranger who happens to be large off the bridge to save the five workers. I know that by doing this he could stop the trolley and save more lives, but I think that killing people is just wrong even if it has good consequences.	Person A (your matching partner) thinks that Zhang Li should push the stranger who happens to be large off the bridge. The reason: I think that Zhang Li should push the stranger who happens to be large to save the five workers. By doing this, he could stop the trolley and save more lives, and I think that it is better to save many lives than just one life.
**No personal force**	A runway trolley is heading down the tracks toward five workers who will all be killed if the trolley proceeds on its present course. Zhang Li is on a footbridge over the tracks, in between the approaching trolley and the five workers. Next to him on this footbridge is a stranger who happens to be large. Zhang Li is near a switch that opens the footbridge’s trap door, on which the tall stranger is standing. The only way to save the lives of the five workers is to hit the switch, which will drop the tall stranger onto the tracks below where his large body will stop the trolley. The stranger will die if Zhang Li does this, but the five workers will be saved. Should Zhang Li hit the switch in order to save the five workers?	Person A (your matching partner) thinks that Zhang Li should not hit the switch to drop the stranger who happens to be large from the bridge. The reason: I think that Zhang Li should not hit the switch to drop the stranger who happens to be large from the bridge to save the workers. I know that by doing this he could stop the trolley and save more lives, but I think that killing people is just wrong even if it has good consequences.	Person A (your matching partner) thinks that Zhang Li should have hit the switch to drop the stranger who happens to be large from the bridge. The reason: I think that Zhang Li should hit the switch to drop the stranger who happens to be large from the bridge to save the workers. By doing this, he could stop the trolley and save more lives, and I think that it is better to save many lives than just one life.

**Table 3:** Bystanders’ Trust Task Investment (M ± SD) Across Experimental Conditions.

	No Personal Force		Personal Force	
	*M*	*SD*	*M*	*SD*
**Non-utilitarian Agent**	6.42	3.10	7.36	2.47
**Utilitarian Agent**	5.18	3.44	3.91	2.93

## Data Availability

The raw data supporting the conclusions of this article will be made available by the authors on request.
